# 1000 collared cementless femoral stems: periprosthetic fracture rates in 500 THA versus 500 hemiarthroplasties

**DOI:** 10.1007/s00590-025-04358-6

**Published:** 2025-06-08

**Authors:** Brian Rigney, Evelyn P. Murphy, Meadhbh Ni Mhiochain De Grae, Christopher Fenelon, Stephen R. Kearns, Fintan Shannon, Colin G. Murphy, Gerard A. Sheridan

**Affiliations:** University Hospital Galway, Galway, Ireland

**Keywords:** Total hip arthroplasty, Hemiarthroplasty, Neck of femur fracture, Collared cementless femoral stems, Periprosthetic fracture

## Abstract

**Introduction:**

This study investigates the claim that the majority of elderly patients undergoing elective total hip arthroplasty (THA) that would routinely be treated with a collared cementless stem are just one fall away from a neck of femur fracture that would then commit them to a cemented femoral prosthesis in many other institutions. This study aims to compare the incidence of intra-operative periprosthetic fracture (PPF) and early postoperative PPF using a cementless collared femoral stem between 500 consecutive elective total hip arthroplasty (THA) patients and 500 consecutive neck of femur (NOF) fracture patients treated with a hemiarthroplasty.

**Methods:**

This was a single-institution retrospective cohort study between August 2015 and December 2023 assessing 500 consecutive NOF fractures and 500 consecutive elective THAs treated with a single brand of collared cementless femoral stem. The primary outcome measure was intra-operative PPF. Secondary outcome measures included early PPF, defined as PPF within 90 days of initial surgery and 90-day mortality.

**Results:**

The mean age in the THA group was 66 (range 32–94) with 246/500 (49.2%) patients being female. In the NOF group, the mean age was 80 (range 45–102) with 350/500 (70%) of patients being female. In the THA group, seven (1.4%) of patients sustained an intra-operative fracture compared with 15 (3%) patients in the NOF group (*p* = 0.09). There were three (0.6%) early PPFs in the elective group compared to four (0.8%) in the NOF group (*p* = 0.7). The 90-day mortality was zero in the elective group and 8.8% in the NOF group. None of the NOF patients with an intra-operative fracture died at the time of follow-up.

**Conclusion:**

Collared cementless femoral stems are safe for use in hemiarthroplasty for hip fracture, especially in an institution that performs cementless HA as standard practice for all hip fractures requiring hemiarthroplasty.

## Introduction

In hip reconstructive surgery, there is ongoing debate as to whether cemented or cementless fixation is optimal for the femoral component. Previous studies have shown an increased PPF rate with cementless components both intra-operatively and in the early postoperative period [1]. On the other hand, cement comes with a longer operating time [2] and the risk of bone cement implantation syndrome (BCIS), which can be fatal [3]. Additionally, recent evidence shows that the incidence of PPF in cemented components is higher than initially reported, due to the open reduction internal fixation procedures being performed in trauma units that were not traditionally reporting into national joint registries, thereby masking outcomes [4].

The benefits of a collar in cementless femoral fixation have been widely reported as it reduces rotation and early subsidence which therefore reduces the risk of periprosthetic fracture (PPF) [5]. In the reporting institution, it is common practice to use collared cementless stems for hemiarthroplasty (HA) procedures for hip fracture surgery. In Ireland as a whole, the majority of surgeons use cemented components for neck of femur (NOF) fracture, with 78% of femoral stems being cemented [6]. This is in contrary to the elective arthroplasty practice, where the majority (61%) of femoral stems are cementless [7]. The reason for higher rates of cementless use in elective THA compared to HA is that surgeons anticipate poorer bone quality in the hip fracture population. For this reason, most guidelines recommend the use of cemented femoral stems in HA surgery.

The hypothesis of the current study is that in an institution where cementless collared femoral stems are used regularly for both HA and THA procedures, the risks of intra-operative and postoperative PPFs will be significantly lower than reported in the general literature. We pose that the majority of elderly patients undergoing elective THA that would be treated with a collared cementless stem are just one fall away from a NOF fracture (that would then commit them to a cemented prosthesis in many institutions). We therefore aim to compare the incidence of intra-operative PPF and early postoperative PPF using a cementless collared femoral stem between 500 consecutive elective total hip arthroplasty (THA) patients and 500 consecutive NOF patients treated with a hemiarthroplasty.

## Methods

**Fig. 1:**
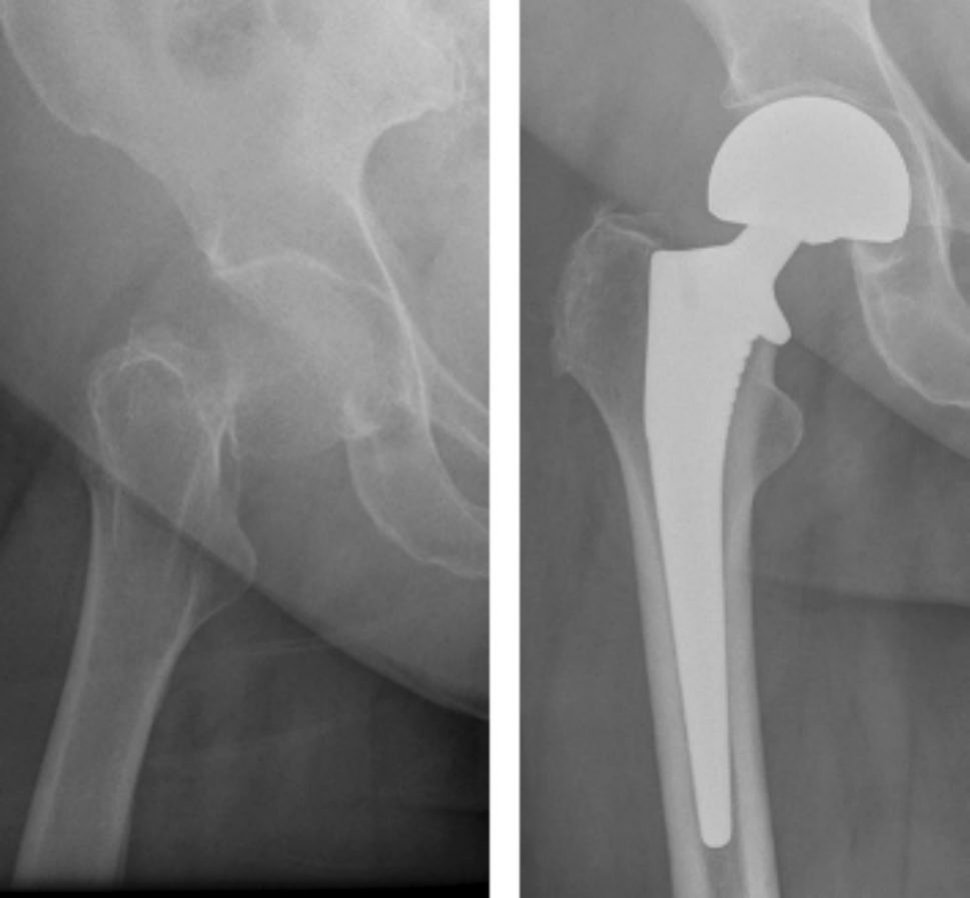
Neck of femur fracture and hemiarthroplasty

**Fig. 2:**
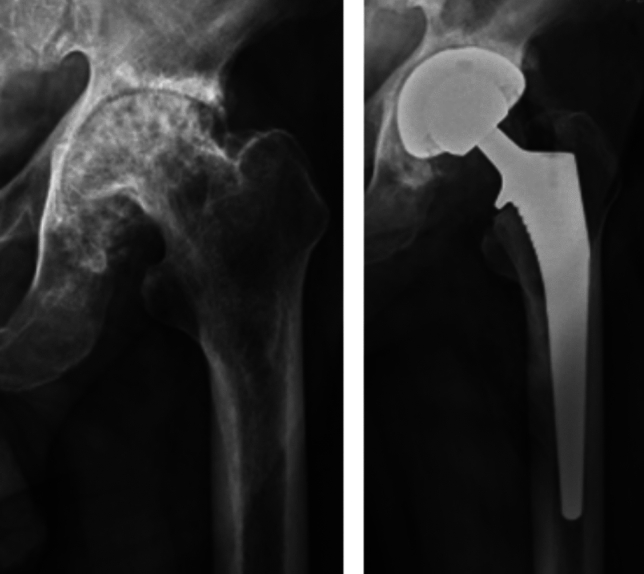
Hip osteoarthritis and total hip arthroplasty

This was a single-institution retrospective cohort study between August 2015 and December 2023 assessing 500 consecutive NOF fractures and 500 consecutive elective THAs treated with a single brand *(Corail®, DePuy Synthes)* of collared cementless femoral stem (Figs. 1 and 2). All cases are performed by consultant surgeons or by surgical trainees supervised by consultant surgeons. All of the THA patients were under the care of a fellowship-trained arthroplasty surgeon, whereas the NOF patients were under the care of the trauma surgeon on call for the day of their operation. The trauma surgeon was not necessarily an arthroplasty-trained surgeon. However, all surgeons performing cementless HA procedures for NOF fractures were performing this procedure on a regular basis on their trauma list.

The inclusion criteria in the NOF group were any patient treated with a hemiarthroplasty using a cementless collared *(Corail®, DePuy Synthes)* stem. Exclusion criteria was pathological NOF fracture or patients treated with a THA. The inclusion criterion in the THA group was any patient treated with a cementless collared *(Corail®, DePuy Synthes)* stem undergoing a primary THA for osteoarthritis. We excluded patients who were having their operation for trauma, tumor or had previous metalwork inserted in their hip.

The primary outcome measure was intra-operative PPF defined as a fracture noted intra-op and the treatment decision made at the time of the fracture being identified. Secondary outcome measures included early PPF, defined as PPF within 90 days of initial surgery (this was to account for any intra-operative PPF that may have been missed), and 90-day mortality.

Data sources used included theater logbooks, the institution’s Electronic Patient Record, the national death register and our local and national radiology databases (AGFA imaging and the National Integrated Medical Imaging System, respectively). Data extracted included demographics, outcomes and date of death. Fractures were analyzed as per Vancouver classification [8]. Radiographs were analyzed by two reviewers to Dorr classification [9]. The senior author arbitrated on any disagreements.

Statistical analysis was performed using Stata/IC 13.1 for Mac (64-bit Intel), with the level of significance set at 0.05. Chi-squared and Fischer exact tests were used for categorical dependent and independent variable data. Two sample t test with equal variance was used to compare interval variables between two groups.

With respect to previous studies, an anticipated 3.7% intra-operative PPF rate in the NOF group was expected and a 0.8% rate in the THA group was expected [2, 4]. It was calculated that at 80% power, an alpha of 0.05 and beta of 0.2, a sample size of 409 in each group would be required to detect a significant difference. This study received ethical approval from our institutional review board.

## Results

The mean age in the THA group was 66 (range 32–94) with 246/500 (49.2%) patients being female. In the NOF group, the mean age was 80 (range 45–102) with 350/500 (70%) of patients being female. In the THA group, 91 patients were classified as Dorr C. In the NOF group, 188 patients were classified as Dorr C (Table 1). All patients had radiographs immediately postoperatively. Of the THA patients, 99% had radiographic follow-up of > 30 days and 95.6% had radiographic follow-up of > 90 days. Of the HA patients, 91.2% had radiographic follow-up > 30 days and 70% had radiographic follow-up of > 90 days.

**Table 1:** Demographics

	THA	HA	
Total patients	500	500	
Mean age (range)	66 (32–94)	80 (45–102)	*p* = < 0.05
Female (%)	246 (49.2%)	350 (70%)	*p* = < 0.05
Dorr A	11	2	*p* = 0.01
Dorr B	398	308	*p* = 0.001
Dorr C	91	188	*p* = 0.001

### Intra-operative fracture

**Fig. 3:**
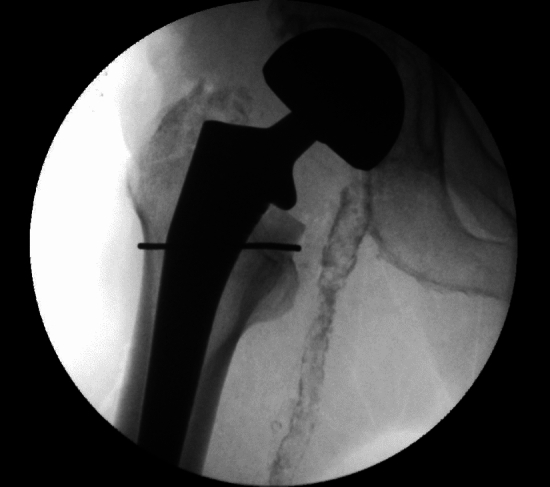
Intra-operative image of hemiarthroplasty with cable

In the THA group, seven (1.4%) of patients sustained an intra-operative fracture compared with 15 (3%) patients in the NOF group (*p* = 0.09). The fracture pattern for all patients as per the Vancouver classification was B2 (undisplaced meta-diaphyseal crack). Table 2 outlines the treatment modalities, with the most commonly employed approach involving the use of a cable (Fig. 3).

**Table 2:** Intra-operative fracture

	THA (*n* = 500)	HA (*n* = 500)	
Intra-operative fracture	7 (1.4%)	15 (3%)	*p* = 0.09
Treatment			
Cable	3 (0.6%)	13 (2.6%)	
Cable and Hook plate	3 (0.6%)	2 (0.4%)	
AP screw	1 (0.2%)		
Pattern			
B2 (undisplaced meta-diaphyseal crack)	7 (1.4%)	15 (3%)	
Dorr classification			
A	0	0	
B	4 (0.8%)	6 (1.2%)	
C	3 (0.6%)	9 (1.8%)

### *Early PPF (*< *90 days)*

**Fig. 4:**
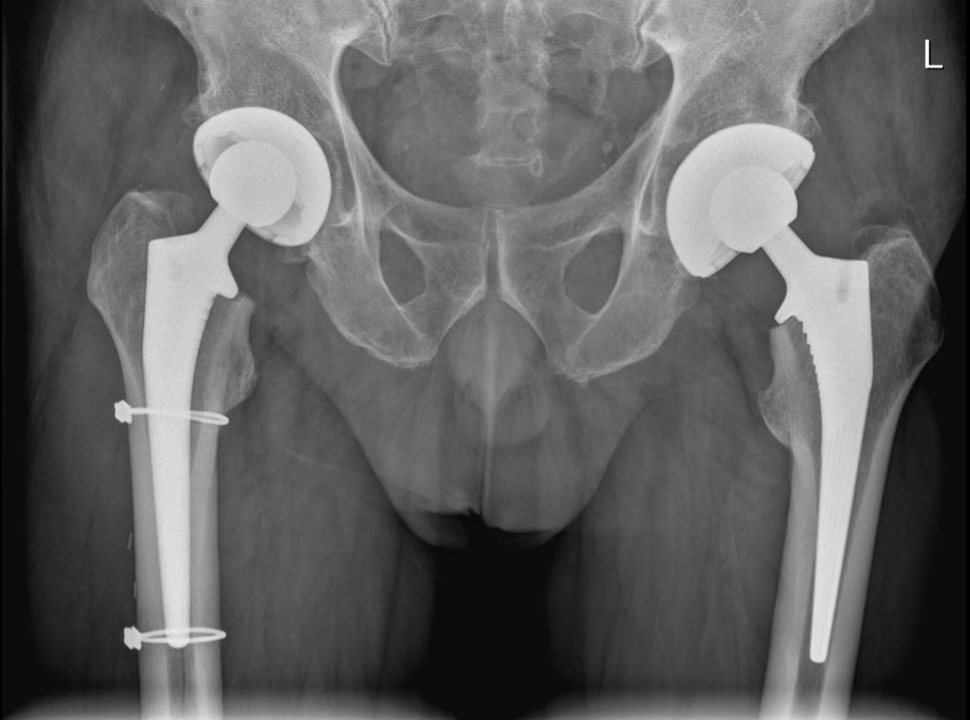
Periprosthetic fracture treated with cables

**Fig. 5:**
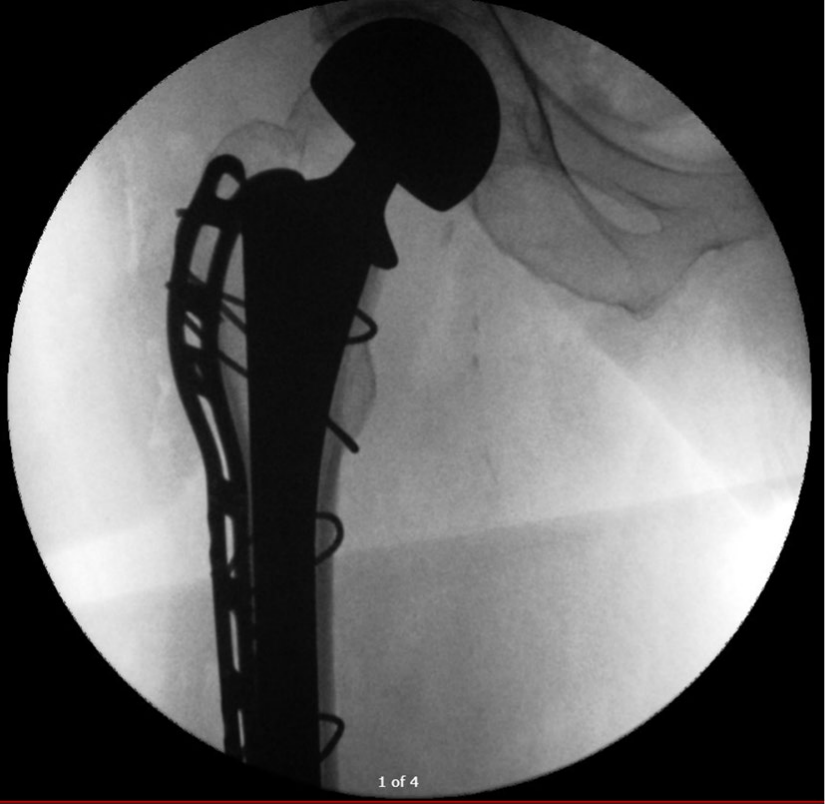
Intra-operative screening of cable and hook plate construct

There were three (0.6%) early PPFs in the elective group compared to four (0.8%) in the NOF group (*p* = 0.7). The respective Vancouver and Dorr classifications are outlined in Table 3. Of the three early in the THA group, two patients fractured following a fall and one patient was found to have an undisplaced Vancouver B fracture. This was felt to be a likely missed intra-operative fracture and was treated with cables alone (Fig. 4). The other two fractures were treated with a cable and hook plate construct. The four patients in the NOF group suffered early PPF following falls. One patient with a Vancouver A fracture pattern was treated non-operatively and was discharged successfully. The other three NOF patients suffered Vancouver B fractures, two of which were treated with a cable and hook plate construct (Fig. 5), and the final patient was revised to a diaphyseal bearing stem. In total, three (0.6%) patients underwent re-operation in each group.

**Table 3:** Early periprosthetic fracture (PPF)

	THA	HA	
Early PPF %	3 (0.6%)	4 (0.8%)	*p* = 0.7
Treatment			
Cable	1	–	
Cable and Hook plate	2	2	
Revision stem	–	1	
Non-operative	–	1	
Pattern			
Vancouver A	1	1	
Vancouver B	1	3	
Vancouver C	1	0	
Dorr classification			
A	0	0	
B	2	2	
C	1	1

### Mortality

The 90-day mortality was zero in the elective group and 44 (8.8%) in the NOF group. None of the NOF patients with an intra-operative fracture died at the time of follow-up. One NOF patient died following an early PPF. This death occurred 55 days following their index surgery (Table 4).

**Table 4:** Mortality

	Elective	Hemi
90-day mortality (all patients) (*N* = 500)	0	44 (8.8%)
90-day mortality (intra-operative fracture)	0 (*n* = 7)	0 (*n* = 15)
90-day mortality (early periprosthetic fracture)	0 (*n* = 3)	1 (*n* = 4)

## Discussion

These results show no statistically significant difference in intra-operative or early PPF rates between the two groups. Additionally, intra-operative PPF did not increase postoperative mortality in the hip fracture cohort or in the elective THA cohort. This study demonstrates that it is safe to use cementless components in the NOF population for HA.

As expected, there was a great deal of heterogeneity between two groups in terms of age, sex and Dorr classification. Additionally, the surgeons operating on the NOF patients were not necessarily arthroplasty specialists. This adds for an interesting discussion point and to the generalizability of the result. Despite the hip fracture patients being older and therefore more likely to be at risk of PPF, as well as the procedures being performed by non-fellowship-trained arthroplasty surgeons, there was no increased risk of PPF. Additionally, the PPF rates observed in our NOF patient cohort are lower than previously reported rates in the literature, which range from 3.7 to 13% [2, 10, 11]. This suggests that a culture of using a single stem type within an institution may improve results and reduce the risk of intra-operative and postoperative PPF rates.

To our knowledge, this is the first study of this kind in the literature to date, comparing the performance of a single collared cementless stem in a hip fracture cohort to a younger elective THA cohort. The hypothesis is supported here—if surgeons are comfortable using cementless collared femoral stems in elective THA procedures (which will often include elderly patients with similar bone quality to HA hip fracture patients), then similar results can be expected with the same stem in a hip fracture patient undergoing HA. The reporting institution previously published on PPF rates in cemented vs cementless hemiarthroplasty, finding that intra-operative calcar fractures did not influence mortality rates [12]. This is in keeping with the findings here with no deaths at 90 days in those who sustained an intra-operative PPF.

Although this study does not compare cement to cementless components, it is still related to the use of cement. The national guidelines in the United Kingdom recommend the use of cemented components for NOF fracture for all patients. However, this recommendation came from 2011, and was based on evidence from elective surgery at that time. As a result, NICE are recommending further research into femoral component design [13]. With the recent evidence from the Lynch Wong et al. showing a 5 times increased risk of postoperative PPF in cemented taper slip stems in elderly men [4], this guidance may be open to change in the future.

### Limitations

The limitations of this study are largely due to its nature, being retrospective. The focus was on intra-operative PPF, and early PPF was recorded largely to account for any missed intra-operative fractures. It is known that NOF patients are generally more comorbid than elective THA patients [14]. In order to give a “real-world” take on this topic, cohorts were not case-matched. All NOF fracture patients who were deemed fit for a THA were excluded from this study. We used Dorr classification as a surrogate marker for bone quality [15]. There was significantly more Dorr C femurs in the NOF group compared to the THR group (188/500 compared with 91/500). Based on the spread of the Dorr classification, the increased age profile and the very nature of hip fractures being a fragility fracture, one can infer that the bone quality was overall worse in the NOF group and that one is largely dealing with an osteoporotic population.

## Conclusion

There is no significant difference in intra-operative or early PPF rates between a frail NOF population receiving a cementless collared stem for hemiarthroplasty and an elective population receiving the same stem for a primary THA. Collared cementless femoral stems are safe for use in hemiarthroplasty, especially in an institution that performs cementless HA as standard practice for all hip fractures requiring hemiarthroplasty. Thus, if a surgeon uses cementless components in their elective practice, they should be able to consider it in their trauma practice to reduce the risks associated with cement.

## Data Availability

No datasets were generated or analyzed during the current study.
